# All-*Trans* Retinoic Acid Promotes an M1- to M2-Phenotype Shift and Inhibits Macrophage-Mediated Immunity to *Leishmania major*

**DOI:** 10.3389/fimmu.2017.01560

**Published:** 2017-11-17

**Authors:** Natália S. Vellozo, Sâmara T. Pereira-Marques, Mariela P. Cabral-Piccin, Alessandra A. Filardy, Flávia L. Ribeiro-Gomes, Thaís S. Rigoni, George A. DosReis, Marcela F. Lopes

**Affiliations:** ^1^Instituto de Biofísica Carlos Chagas Filho, Universidade Federal do Rio de Janeiro, Rio de Janeiro, Brazil; ^2^Instituto de Microbiologia Paulo de Góes, Universidade Federal do Rio de Janeiro, Rio de Janeiro, Brazil; ^3^Instituto Oswaldo Cruz, Fundação Oswaldo Cruz, Rio de Janeiro, Brazil; ^4^Instituto Nacional para Pesquisa Translacional em Saúde e Ambiente na Região Amazônica, Conselho Nacional de Desenvolvimento Científico e Tecnológico, Rio de Janeiro, Brazil

**Keywords:** all-*trans* retinoic acid, alternatively activated macrophage, classically activated macrophage, Leishmaniasis, nitric oxide, parasite infection, retinoic acid, vitamin A

## Abstract

As key cells, able to host and kill *Leishmania* parasites, inflammatory monocytes/macrophages are potential vaccine and therapeutic targets to improve immune responses in Leishmaniasis. Macrophage phenotypes range from M1, which express NO-mediated microbial killing, to M2 macrophages that might help infection. Resistance to Leishmaniasis depends on *Leishmania* species, mouse strain, and both innate and adaptive immunity. C57BL/6 (B6) mice are resistant and control infection, whereas *Leishmania* parasites thrive in BALB/c mice, which are susceptible to develop cutaneous lesions in the course of infection with *Leishmania major*, but not upon infection with *Leishmania braziliensis*. Here, we investigated whether a deficit in early maturation of inflammatory monocytes into macrophages in BALB/c mice underlies increased susceptibility to *L. major* versus *L. braziliensis* parasites. We show that, after infection with *L. braziliensis*, monocytes are recruited to peritoneum, differentiate into macrophages, and develop an M1 phenotype able to produce proinflammatory cytokines in both B6 and BALB/c mice. Nonetheless, more mature macrophages from B6 mice expressed inducible NO synthase (iNOS) and higher NO production in response to *L. braziliensis* parasites, whereas BALB/c mice developed macrophages expressing an incomplete M1 phenotype. By contrast, monocytes recruited upon *L. major* infection gave rise to immature macrophages that failed to induce an M1 response in BALB/c mice. Overall, these results are consistent with the idea that resistance to *Leishmania* infection correlates with improved maturation of macrophages in a mouse-strain and *Leishmania*-species dependent manner. All-*trans* retinoic acid (ATRA) has been proposed as a therapy to differentiate immature myeloid cells into macrophages and help immunity to tumors. To prompt monocyte to macrophage maturation upon *L. major* infection, we treated B6 and BALB/c mice with ATRA. Unexpectedly, treatment with ATRA reduced proinflammatory cytokines, iNOS expression, and parasite killing by macrophages. Moreover, ATRA promoted an M1 to M2 transition in bone marrow-derived macrophages from both strains. Therefore, ATRA uncouples macrophage maturation and development of M1 phenotype and downmodulates macrophage-mediated immunity to *L. major* parasites. Cautions should be taken for the therapeutic use of ATRA, by considering direct effects on innate immunity to intracellular pathogens.

## Introduction

*Leishmania* parasites infect macrophages and cause Leishmaniasis, ranging from localized cutaneous or mucocutaneous to visceral or disseminated diseases. Monocytes/macrophages, as host and effector cells, play a major role to fight *Leishmania* infection in innate immunity, as well as an effector arm of adaptive immunity, upon activation by cytokines produced by T lymphocytes. IFN-γ-producing Th1 cells activate macrophages to express inducible NO synthase (iNOS/NOS2) and effect NO-mediated killing of *Leishmania* parasites. By contrast, Th2 cytokines, such as IL-4 and IL-10, inhibit macrophage activation, induce arginase expression, and promote parasite infection (1–6). By analogy to Th1 and Th2 lymphocytes, the functional phenotypes of macrophages are known as M1 and M2 or as classically activated and alternatively activated macrophages, with a range of intermediates between the extreme IFN/LPS and IL-4-induced phenotypes (7–11).

While Th1/M1 responses mediate immunity to *Leishmania* parasites in resistant C57BL/6 (B6) mice, Th2/M2 cells underlie susceptibility to *Leishmania major* infection in BALB/c mice, although the role of IL-4 on T cells and macrophages remains controversial (1, 5, 12–16). Equally important is the notion that exacerbated type-1 immune responses contribute for pathology in mucocutaneous Leishmaniasis, upon infection with *Leishmania braziliensis* (17, 18). In line with this, there are still unsolved issues, which once elucidated might help development of vaccines and therapies to improve immunity to *Leishmania* infection and/or prevent pathology. For instance, macrophages from resistant or susceptible strains can express features associated with M1 or M2 phenotypes even in the absence of adaptive immunity (7). Likewise, earlier differentiation kinetics of F4/80^+^ monocytic cells in B6 mice versus a predominance of more immature cells in BALB/c mice may also contribute to development of resistance versus susceptibility to *L. major* infection (19–21). Moreover, *L. major* infection in B6 mice induces early inflammatory monocytes (CD11b^+^Ly6C^+^F4/80^int^ cells), which already express ROS or NO-mediated killing (22, 23). Therefore, the differentiation of immature monocytic cells into effector monocytes/macrophages is a promising vaccine/therapeutic target in Leishmaniasis.

All-*trans* retinoic acid (ATRA) is a vitamin A active metabolite, which binds to intracellular receptors in immune cells and may affect innate and adaptive immunity (24, 25). ATRA promotes differentiation of immature myeloid cells (IMCs) into macrophages and has been considered to promote anti-tumor immunity, by targeting IMC suppression of CD8 T-cell-mediated immunity (26). We have previously shown that inflammatory monocytes express features of IMCs upon *L. major* infection and that treatment with ATRA prevents NO-mediated suppression of T-cell proliferation in lymph nodes from infected B6 mice (23). However, ATRA-treated mice developed increased footpad lesions and parasite load in draining lymph nodes (23). We hypothesized that ATRA might directly affect macrophage phenotype and macrophage-mediated immunity to *L. major*. We also considered that treatment with ATRA could counteract the maturation deficit in monocytes/macrophages from BALB/c mice.

Here, we investigated whether a deficient maturation of inflammatory monocytes into macrophages may underlie increased susceptibility to *L. major* versus *L. braziliensis* parasites in BALB/c mice. *L. braziliensis*, but not *L. major* infection induced inflammatory monocytes that mature into macrophages and expressed an M1 phenotype. We also show that treatment with ATRA *in vivo* negatively affected the functional phenotype of inflammatory monocytes/macrophages and immunity to *L. major* infection. Furthermore, ATRA prevented induction of effector M1 macrophages, by promoting an M1- to M2-phenotype shift in bone marrow-derived macrophages (BMDMs) from both B6 and BALB/c mice.

## Materials and Methods

### Animals and Parasites

C57BL/6 and BALB/c mice were obtained from the Oswaldo Cruz Foundation (FIOCRUZ, Rio de Janeiro, Brazil) and maintained in the animal facility at the Federal University of Rio de Janeiro (UFRJ). All experiments were approved and conducted in accordance with guidelines of the Ethics Committee for Use of Animals (UFRJ) (Protocol no. 078/16). We used the following parasite strains: the Venezuelan isolate Torres of *L. braziliensis* (27), *L. major* LV39 (MRHO/Sv/59/P), or a stable transfected line of *L. major* Friedlin FV1 (MHOM/IL/80/FN), which express a red fluorescent protein (*Lm*-RFP) (28). Parasites were isolated from popliteal lymph nodes of infected BALB/c mice and maintained up to 4 weeks at 28°C in Schneider’s medium (Sigma, USA), supplemented with 2% of sterile human urine, 2 mM of l-glutamine, 10 µg/mL of gentamicin, and 10% of fetal bovine serum (FBS, Gibco BRL, South America). For infection, *Leishmania* parasites were cultured until stationary phase at 28°C in Schneider’s medium. *L. braziliensis* parasites were then purified in a 10% ficoll gradient to obtain metacyclic forms (29).

### *Leishmania* Infection

Female B6 and BALB/c mice, aging 6–8 weeks, were infected i.p. with 3 × 10^6^ promastigote parasites of *L. major* LV39 or *Lm*-RFP or with 6 × 10^5^ metacyclic forms of *L. braziliensis*.

### Administration of ATRA *In Vivo*

C57BL/6 and BALB/c mice were infected i.p. with *L. major*. Upon 24 h, infected mice were injected i.p. with 100 µL of a 100-µM solution of ATRA (Sigma, St Louis, MO, USA) or control vehicle (0.2% dimethyl-sulfoxide, DMSO, Sigma). After 24 h, peritoneal exudates were collected for analysis and cultures.

### Peritoneal Exudate Cells (PECs)

Peritoneal exudate cells were collected in 4 mL of DMEM (Invitrogen Life Technologies), supplemented with 2-mM glutamine, 5 × 10^5^ M 2-ME, 10-µg/mL gentamicin, 1-mM sodium pyruvate, and 0.1-mM MEM non-essential amino acids (culture medium). Upon centrifugation, supernatants were collected for cytokine assays and NO production. PECs were processed for flow cytometry or cultured and infected, as bellow. After 72 h, culture supernatants were collected for cytokine and NO assays, and cultured cells were used to determine parasite burden.

### Macrophage Infection and Parasite Load

Peritoneal exudate cells from control mice or from mice infected with *L. major* LV39, *Lm*-RFP, or *L. braziliensis* were cultured in triplicates at 5 × 10^5^ cells/well in 48-well vessels or at 1 × 10^6^/well in 24-well plates during 1 h, and then washed for removal of non-adherent cells. Adherent macrophages were reinfected (for 4 h) with 3 × 10^6^ *L. major* or *Lm*-RFP promastigotes or with 4 × 10^4^ metacyclic forms of *L. braziliensis*. Cultures were washed for removal of extracellular parasites and additional non-adherent cells. Infected macrophages were maintained in culture medium plus 10% FBS at 37°C and 7% CO_2_ for 3–4 days. For evaluation of *Lm*-RFP infection, macrophages were detached from 24-well plates and analyzed by flow cytometry. In cultures established in 48-well vessels, supernatants were collected for cytokine analyses and replaced by Scheneider’s medium. Macrophages were further cultured for at least 3 days at 28°C, in a BOD incubator (Cienlab) for determination of parasite load. Parasites released in culture supernatants were then counted in a Beckman Coulter (USA) within a range of 3–6 µm, for exclusion of cells.

### BMDM Differentiation and Treatment with ATRA *In Vitro*

Tibiae from B6 and BALB/c mice (5–8 weeks) were removed and washed with HBSS (Gibco) plus 2% of FBS. Cells were collected, washed, and cultured at 5 × 10^4^/well in 48-well plates or at 1 × 10^6^/well in 24-well vessels for flow cytometry. Cultures were maintained for 7 days in culture medium plus 10% FBS, 20% of the L929 cell culture supernatant, as an M-CSF source, following a protocol adapted from Sutterwala et al. (30). After 7 days, cultures were treated with IFN-γ (0.5 ng/mL, R&D Systems) for 24 h. Then, cells were washed and cultured in the presence of IL-4 (5 ng/mL, R&D Systems), as before (31). DMSO (0.004%) or ATRA (2 µM) were also added to cultures. Three days later, cells were treated or not with 1 µg/mL of LPS from *Salmonella enterica* serovar Typhimurium (Sigma) and cultured for further 3 days. Culture supernatants were evaluated for cytokine and NO production and cells were prepared for flow cytometry.

### Flow Cytometry

Peritoneal exudate cells or cultured cells were washed in FACS buffer (plus 2% FBS), followed by incubation with anti-CD16/CD32 (eBioscience, San Diego, CA, USA) for Fc blocking. We stained cells with anti-CD11b, anti-F4/80, and anti-Ly6C (HK1.4) labeled with PE, FITC or allophycocyanin (BD Biosciences, Chicago, IL, USA), or with Alexa Fluor 488-labeled anti-CD301 (MGL) mAb (AbD Serotec, Kidlington, UK), or control rat IgG2a mAb (R&D Systems, Minneapolis, MN, USA). For intracellular staining, we washed, permeabilized, and stained cells with PE-labeled anti-IL-12p35 or control murine IgG1 mAb (R&D Systems), anti-NOS2 or control rat IgG2a mAb (BD Bioscience), or with FITC-labeled anti-arginase 1 or control sheep IgG mAb. Cells were washed, fixed, and acquired with the CellQuest software, on a FACSCalibur system (BD Biosciences). For analysis, we used the FlowJo software (TreeStar, Ashland, OR, USA). For IL-12p35^+^, NOS2^+^ (M1), and CD301^+^, arginase^+^ (M2) subsets, gates were based on the exclusion of background staining with isotype control mAbs, as in Figure 2B. For evaluation of *Lm*-RFP infection, PECs or cultured macrophages were first stained with allophycocyanin-anti-F4/80 and then analyzed for *Lm*-RFP^+^ cells within gated F4/80^+^ macrophages.

### Cytokine and Chemokine Assays

Supernatants from cultures or peritoneal exudates were used for detection of cytokines (IL-10, IL-12p70, G-CSF, TNF-α) and chemokines (CCL17, CXCL9, CXCL13) by ELISA assays. For that, we used pairs of specific mAbs (R&D Systems or eBioscience), one of which was labeled with biotin, and then developed with streptavidin-alkaline phosphatase (Invitrogen Life Technologies) and p-nitrophenyl phosphate (Thermo Scientific Pierce, Waltham, MA, USA) as substrate, according to manufacturer’s guidelines.

### Nitric Oxide

Production of NO was determined indirectly by quantification of nitrites. Peritoneal exudates or culture supernatants were mixed with Griess reagent (1% sulfanilamide, 0.1% naphthylethylenediamine dihydrochloride, 2% H3PO4; Sigma) in a 1:1 ratio. A standard curve with known concentrations of sodium nitrite (NaNO_2_) was used and the results were expressed in µM. The optical density was determined at 540 nm on a plate spectrophotometer (VersaMax, Molecular Devices).

### Statistics

All tests were performed by using the GraphPad Prism (v. 6.0). Results are expressed as mean and SEM in figures. The number (*N*) of animals per group is indicated in figure legends. For parasite load, data were transformed to log of parasites per mL for statistical analysis. Data were analyzed by Kolmogorov–Smirnov test for assessing normal distribution and by unpaired Student’s two-tailed *t*-test or ANOVA, followed by Dunnett, Bonferroni, or Tukey post-test. Significant differences are indicated for *P* < 0.05 (*), *P* < 0.01 (**), *P* < 0.001 (***), and *P* < 0.0001 (****). For *in vitro* experiments, data are expressed as the mean of technical replicates per treatment and SEM. Significant differences in *t*-tests are indicated as above.

## Results

### ATRA Induces a Shift from M1 to M2 Phenotype in BMDMs

To address how ATRA affects both M1- and M2-macrophage phenotypes, we cultured BMDMs from B6 and BALB/c mice with a mix of M1/M2-inducing reagents. BMDMs were generated and primed with IFN-γ: BMs from B6 and BALB/c render 90% of macrophages expressing F4/80 at higher levels (F4/80^hi^) upon treatment with IFN-γ. Then, macrophages were cultured with IL-4, followed by LPS stimulation, as before (31), to give identical M1 and M2 conditions to B6 and BALB/c macrophages. BMDMs were first treated or not with ATRA during 3 days (concomitant with IL-4 and before stimulation with LPS) to address ATRA effects during the development of M1/M2 phenotypes, by mimicking stimuli from infection environment.

**Figure 1:**
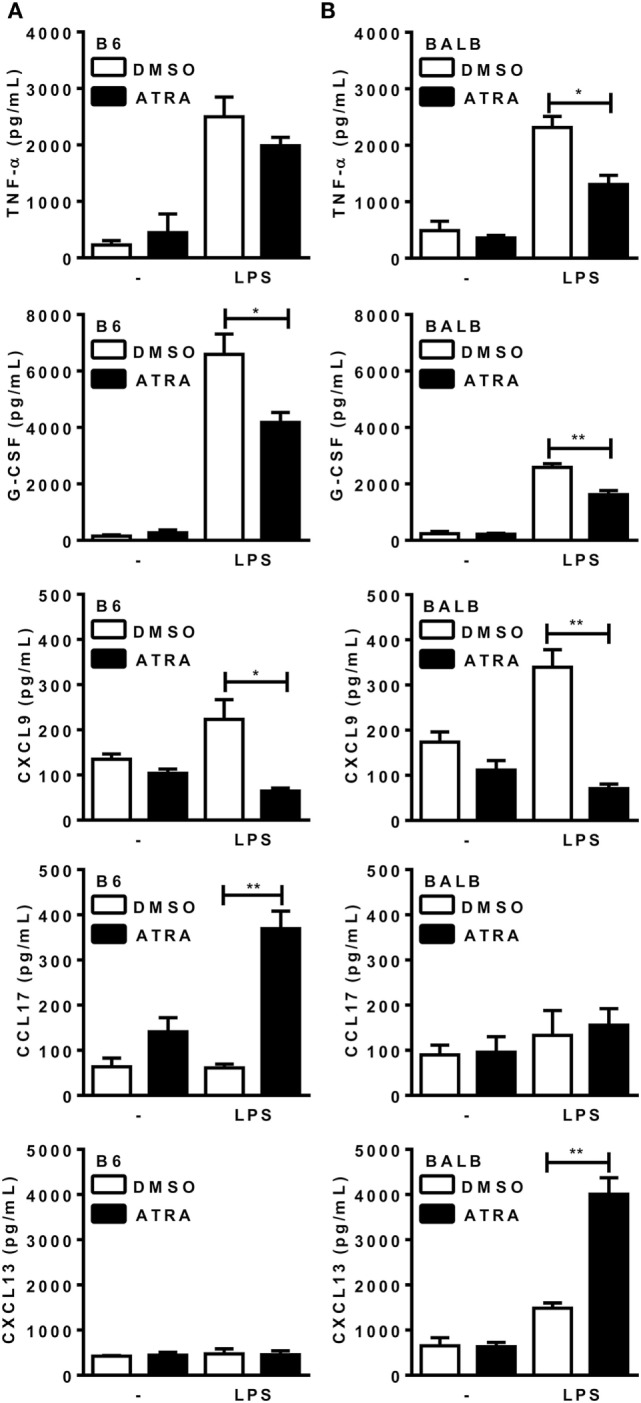
All-*trans* retinoic acid (ATRA) induces bone marrow-derived macrophages (BMDMs) to shift from M1 to M2 macrophages. **(A,B)** BMDMs from B6 and BALB/c mice were primed with IFN-γ (for 24 h). Cells were then treated with IL-4 and ATRA or control vehicle (DMSO) during 3 days and cultured with or without LPS for further 3 days. Cytokines and chemokines in supernatants from **(A)** B6 and **(B)** BALB/c cultures were assayed by ELISA. Cultures were performed in triplicates. Results are expressed as mean and SEM. Significant differences between LPS-activated macrophages treated with or without ATRA were analyzed by *t*-test and indicated for *P* < 0.05 (*) and *P* < 0.01 (**). Data are representative of two independent experiments.

Under these M1/M2 mixed conditions, macrophages from B6 mice secreted M1 cytokines and chemokine, such as TNF-α, G-CSF, and CXCL9 (Figure 1A), and expressed IL-12p35 (not shown), but produced low levels of the M2 chemokines CCL17 and CXCL13. BMDMs from BALB/c mice (Figure 1B), however, produced both M1 and M2 cytokines and chemokines upon consecutive stimulation with IFN-γ, IL-4, and LPS. B6 or BALB/c macrophages did not produce IL-10 under these conditions (not shown). Treatment with ATRA prevented induction of M1 cytokines and CXCL9, but increased M2 chemokines, such as CCL17 in B6 macrophages, as well as CXCL13 in BALB/c macrophages (Figures 1A,B). These results indicate that ATRA promotes an M1- to M2-phenotype shift in BMDMs from mouse strains, either resistant or susceptible to *L. major* infection.

### ATRA Reduces NO Production and NOS2 Expression by BMDMs

**Figure 2:**
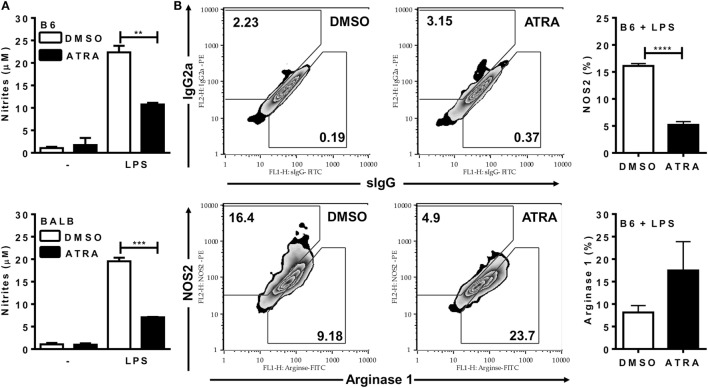
All-*trans* retinoic acid (ATRA) reduces NO production and NOS2 expression by bone marrow-derived macrophages (BMDMs). **(A,B)** BMDMs from B6 and BALB/c mice were primed with IFN-γ (for 24 h). Cells were then treated with IL-4 and ATRA or control vehicle (DMSO) during 3 days and cultured with or without LPS for further 3 days. **(A)** Culture supernatants were collected and tested for NO production by the Griess method. **(B)** F4/80^+^ macrophages from B6 mice were evaluated for NOS2 and arginase expression (lower panels), and for staining with the respective control mAbs (upper panels). Cultures were performed in triplicates. Results are expressed as mean and SEM. Significant differences between LPS-activated macrophages treated with or without ATRA were analyzed by *t*-test and indicated for *P* < 0.01 (**), *P* < 0.001 (***), and *P* < 0.0001 (****). Results depicted in **(A)** are representative of four independent experiments.

Next, we addressed whether ATRA directly affects M1 mechanisms of parasite killing. ATRA inhibited NO production by BMDMs from both B6 and BALB/c mice (Figure 2A). Moreover, treatment with ATRA reduced NOS2 expression and the ratio of NOS2/arginase 1 expression (Figure 2B and not shown) in BMDMs from B6 mice.

### Earlier Monocyte Differentiation to Macrophages in B6 PECs

**Figure 3:**
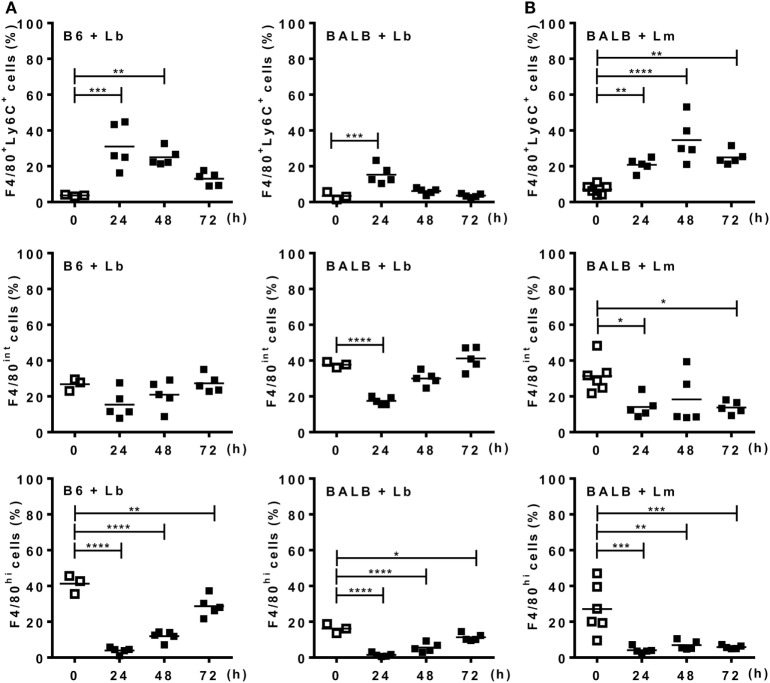
Monocytes differentiate into macrophages upon *Leishmania* infection. Peritoneal exudate cells (PECs) from control [**(A)**, left panels] B6 or **(A,B)** BALB/c mice, as well as from mice infected with **(A)** *L. braziliensis* or **(B)** *L. major* were evaluated for expression of Ly6C and F4/80 within CD11b^+^ gated cells. **(A,B)** Percentages of F4/80^+^Ly6C^+^, F4/80^int^, and F4/80^hi^ cells from **(A)** *L. braziliensis*-infected B6 or BALB/c mice or **(B)** *L. major*-infected BALB/c mice compared with PECs from uninfected (d0) mice. Each symbol represents individual control (□, *N* = 3 or 6 mice/group) or infected mouse (■, *N* = 5 mice/group). Means are represented. Significant differences were analyzed by ANOVA with Dunnett post-test and indicated for *P* < 0.05 (*), *P* < 0.01 (**), *P* < 0.001 (***), and *P* < 0.0001 (****).

Before evaluating ATRA effects on the phenotype of inflammatory monocytes/macrophages recruited by *Leishmania* parasites, we investigated whether there is a differential kinetics of monocyte recruitment and differentiation in B6 versus BALB/c mice upon i.p. infection with *L. braziliensis* (Lb) or *L. major* (Figure 3; Figures S1 and S2 in Supplementary Material). To follow macrophage differentiation *in vivo*, we first infected mice with Lb (Figure 3A; Figure S1A in Supplementary Material). Lb infection in both B6 and BALB/c mice quickly recruited CD11b^+^Ly6C^+^F4/80^+^ cells, which decreased thereafter (Figure 3A). Most of these Ly6C^+^ immature monocytes/macrophages expressed intermediate levels of F4/80 (F4/80^int^; not shown). By contrast, proportions (Figure 3A) and numbers (Figure S1A in Supplementary Material) of more mature macrophages, which do not express Ly6C, but are CD11b^+^F4/80^+^ (hi or int) cells, first decreased and then progressively increased in B6 and BALB/c mice. It is noteworthy that F4/80^int^ macrophages predominated over the more mature F4/80^hi^ macrophages in BALB/c, but not B6 mice. BALB/c and B6 mice also recruited CD11b^+^Ly6C^+^F4/80^+^ macrophages upon i.p. infection with *L. major*, by peaking at 48 h (Figure 3B; Figures S1B and S2A in Supplementary Material). There was a decrease in resident F4/80^int^ or F4/80^hi^ macrophages upon 24 or 48 h (Figure 3B; Figure S2C in Supplementary Material), but mature macrophages expressing F4/80 at intermediate or high levels did not change in BALB/c mice thereafter (Figure 3B). These results indicate that more immature macrophages predominate in the peritoneum of BALB/c mice upon infection with *L. major* than after infection with *L. braziliensis*, and also corroborate a previously reported deficit in the differentiation of macrophages from BALB/c mice (19–21).

### Mature Macrophages Express an M1 Phenotype upon Infection with *L. braziliensis*

**Figure 4:**
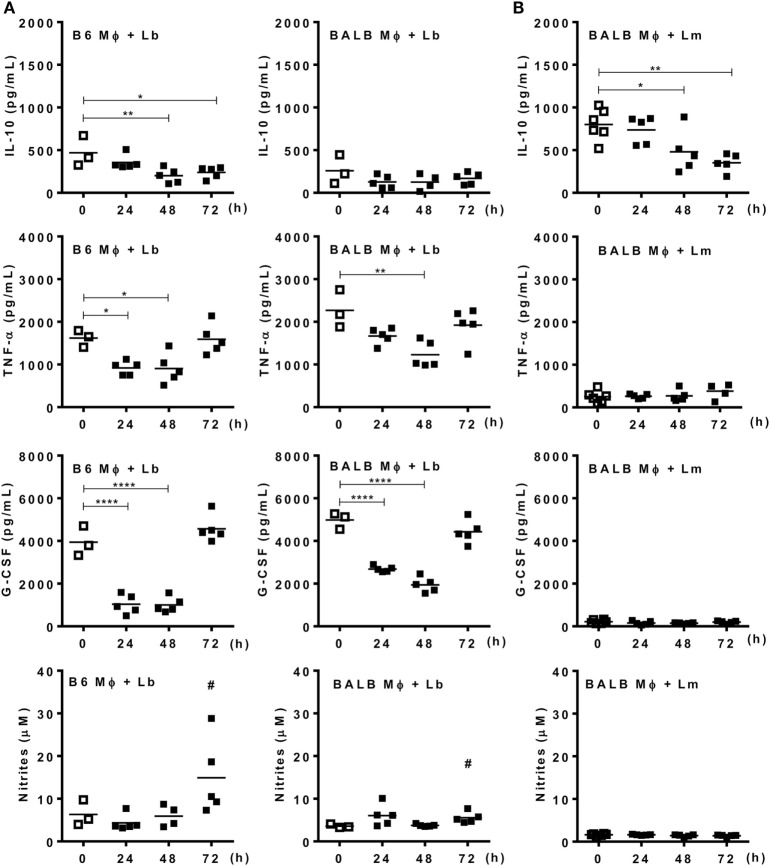
Mature macrophages express an M1 phenotype upon infection with *Leishmania braziliensis*. B6 and BALB/c mice were infected i.p. with **(A)** *L. braziliensis* or **(B)** *L. major*. **(A,B)** Adherent peritoneal exudate cells were reinfected with *L. braziliensis* **(A)** or *L. major* **(B)**. **(A,B)** Culture supernatants were evaluated for the presence of type-1 (TNF-α and G-CSF) and type-2 (IL-10) cytokines by ELISA, and for NO by the Griess method. Each symbol represents individual control (□, *N* = 3 or 6 mice/group) or infected mouse (■, *N* = 5 mice/group). Means are represented. Significant differences were analyzed by ANOVA with Dunnett post-test and indicated for *P* < 0.05 (*), *P* < 0.01 (**), and *P* < 0.0001 (****). In **(A)**, # indicates a difference (*P* < 0.05) between Lb-infected B6 and BALB/c mice, as assessed by *t*-test.

**Figure 5:**
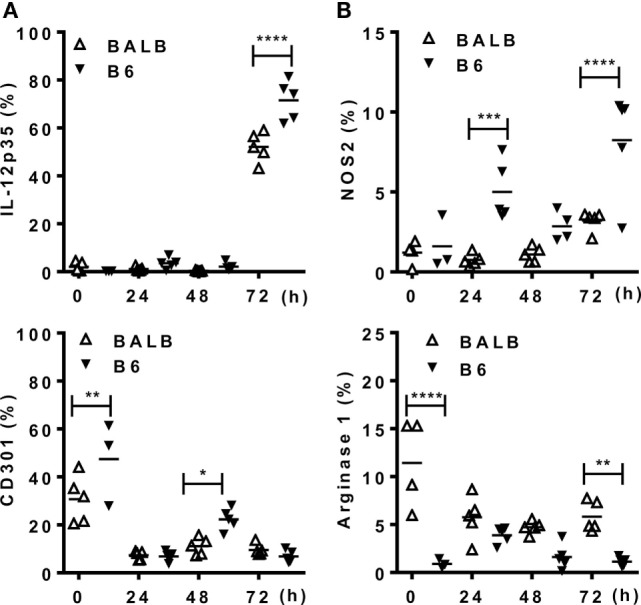
Mature macrophages express M1, but not M2 markers upon infection with *Leishmania braziliensis*. B6 and BALB/c mice were infected i.p. with *L. braziliensis*. **(A,B)** CD11b^+^ peritoneal exudate cells (PECs) were evaluated for the expression of **(A)** IL-12p35 and CD301 (MGL) or for **(B)** NOS2 and arginase 1. Symbols represent PECs from individual BALB/c (Δ) or B6 (▾) mice (*N* = 3–5/group of control mice and *N* = 5 mice/infected group). Means are represented. Significant differences were analyzed by ANOVA with Bonferroni post-test and indicated for *P* < 0.05 (*), *P* < 0.01 (**), *P* < 0.001 (***), and *P* < 0.0001 (****).

**Figure 6:**
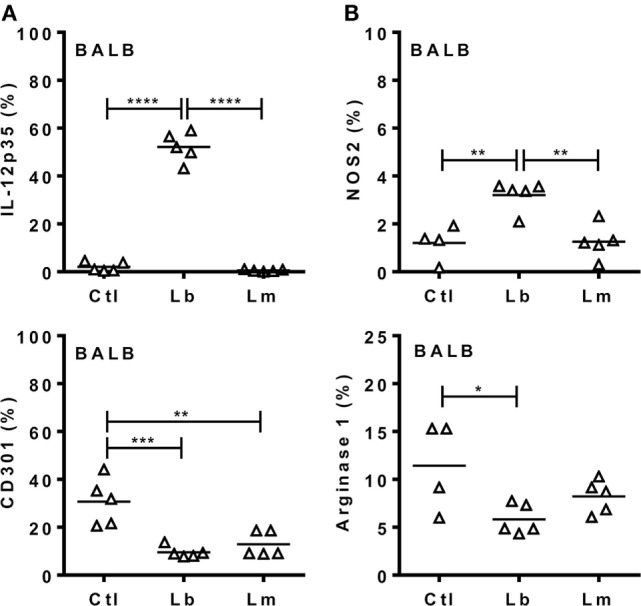
*Leishmania braziliensis*, but not *L. major* induces an M1 response by macrophages from BALB/c mice. BALB/c mice were infected i.p. with *L. braziliensis* or *L. major*. **(A,B)** After 72 h, CD11b^+^ peritoneal exudate cells (PECs) were evaluated for the expression of **(A)** IL-12p35 and CD301 (MGL) or for **(B)** NOS2 and arginase 1. Symbols represent PECs from individual BALB/c (Δ) mice (*N* = 4–5/group of control mice and *N* = 5 mice/infected group). Means are represented. Significant differences were analyzed by ANOVA with Tukey post-test and indicated for *P* < 0.05 (*), *P* < 0.01 (**), *P* < 0.001 (***), and *P* < 0.0001 (****).

Next we addressed the profile of cytokines produced by PECs from mice infected with Lb or *L. major* upon culture and stimulation with *Leishmania* parasites (Figure 4). At 72 h upon i.p. infection with Lb, macrophages from both B6 and BALB/c mice expressed an inflammatory phenotype, by producing TNF-α, G-CSF, and NO, but not IL-10 in Lb-stimulated cultures (Figure 4A). However, more mature macrophages from B6 mice (as in Figure 3A) showed increased NO production compared with less mature macrophages from BALB/c mice (Figure 4A). Interestingly, while Lb also elicited a strong M1 response by resident (d0) macrophages from both B6 and BALB/c mice (Figure 4A), peritoneal macrophages from BALB/c produced *L. major*-induced IL-10, but not inflammatory cytokines (Figure 4B). BALB/c macrophages recruited upon *L. major* infection also failed to produce M1 cytokines or NO upon stimulation with *L. major* (Figure 4B). We conducted parallel analyses to follow the development of M1/M2 phenotypes by flow cytometry upon i.p. infection with *Leishmania* parasites. CD11b^+^ PECs from Lb-infected B6 and BALB/c mice expressed IL-12p35 and NOS2, but not the M2 marker CD301 (MGL) or arginase 1 (Figures 5A,B). Furthermore, expression of IL-12 and NOS2 by macrophages reached higher levels in Lb-infected B6 mice (Figure 5). In contrast to Lb, *L. major* infection did not induce expression of IL-12 or NOS2 in BALB/c macrophages (Figures 6A,B). Therefore, Lb infection induces a stronger M1 response (Figures 4 and 5) by more mature macrophages (Figure 3) from B6 versus BALB/c mice. These results also show that *L. major* is a weaker stimulus to induce monocyte-to-macrophage differentiation (Figure 3) and M1 responses (Figures 4 and 6) as compared with Lb parasites.

### ATRA Reduces M1 Responses and Promotes *L. major* Infection

**Figure 7:**
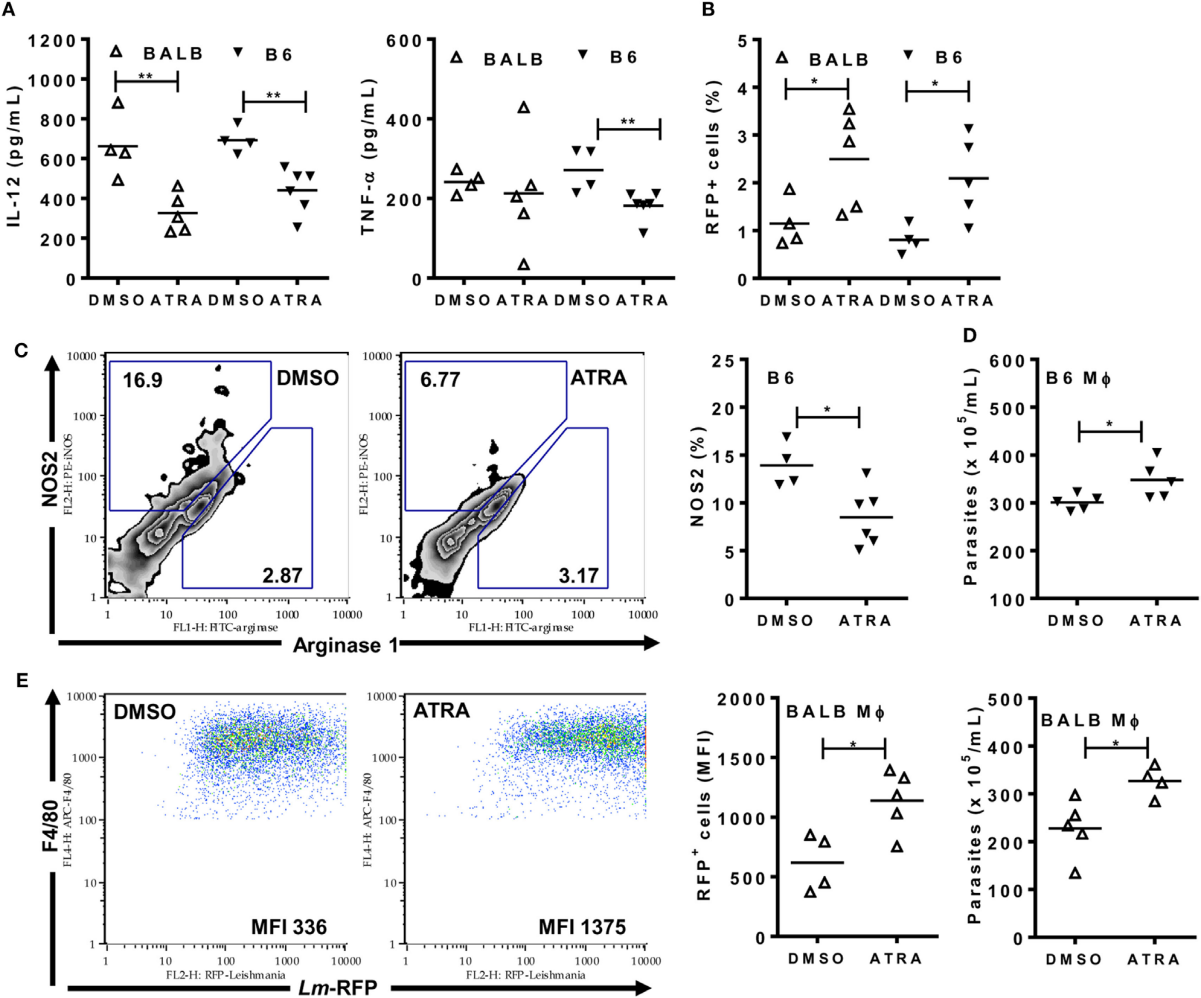
All-*trans* retinoic acid (ATRA) reduces M1 responses and immunity to *Leishmania major* infection. BALB/c and B6 mice were first infected **(A,C,D)** with *L. major* LV39 strain or **(B,E)** with Lm-RFP and then treated with DMSO or ATRA after 24 h. **(A)** Peritoneal exudates were evaluated 48 h upon infection with *L. major* for type-1 cytokines (IL-12 and TNF-α) by ELISA. **(B)** F4/80^+^ peritoneal exudate cells (PECs) were analyzed for the percentages of cells infected with Lm-RFP. **(C)** PECs from infected B6 mice were stained and analyzed for the expression of NOS2 and arginase 1 within F4/80^+^ gated cells. **(D,E)** PECs from infected B6 and BALB/c mice were reinfected **(D)** with *L. major* LV39 or **(E)** with Lm-RFP and parasite burden was **(D)** counted in supernatants or **(E)** determined as Lm-RFP infection within F4/80^+^ cells. MFI stands for median of fluorescence intensity of Lm-RFP-infected macrophages. Symbols represent individual infected (Δ) BALB/c and (▾) B6 mice treated with DMSO (*N* = 4–5 mice/group) or ATRA (*N* = 4–6 mice/group). Means are represented. Significant differences between mice treated with DMSO and ATRA were analyzed by *t*-test and indicated for *P* < 0.05 (*) and *P* < 0.01 (**). Data are representative of two independent experiments.

We used the *L. major* model to test how ATRA affects the functional phenotype of inflammatory monocytes/macrophages from BALB/c and B6 mice (Figures 7A–E). Mice were infected i.p. and treated with ATRA 24 h upon infection. PECs were collected for analyses 24 h after treatment (48 h post-infection). Treatment with ATRA reduced the release of IL-12, TNF-α (Figure 7A), and NO (not shown) in the peritoneum. In addition, ATRA negatively affected NOS2 expression in F4/80^+^ macrophages from B6 mice (Figure 7C). Next, we addressed how treatment with ATRA *in vivo* impacts on the rate of macrophage infection with Lm-RFP parasites (Figure 7B). Although only 1–2% of F4/80^+^ macrophages show Lm-RFP infection (not shown), treatment with ATRA increased Lm-RFP-infected macrophages in both BALB/c and B6 mice (Figure 7B). Macrophages from *L. major-*infected and treated mice were also reinfected with *L. major in vitro* to test their ability to kill the parasites (Figures 7D,E). Upon *in vivo* treatment with ATRA, macrophages were less efficient to control *L. major* infection compared with macrophages from DMSO-treated mice, as evaluated by increased numbers of parasites released in culture supernatants (Figure 7D) or by Lm-RFP infection within macrophages (Figure 7E). Therefore, ATRA negatively affects M1 responses and undermines macrophage-mediated immunity to *L. major* in both resistant B6 and susceptible BALB/c mice.

## Discussion

Treatment with ATRA is considered as a promising therapy to improve immunity against tumors, by inducing differentiation of suppressor monocytes/IMCs into macrophages (26). Nonetheless, ATRA might also affect macrophage phenotype and ability to fight parasites and other pathogens.

It has been reported that inflammatory monocytes are major host and effector cells in *L. major* infection (22, 23, 32) and that delayed differentiation of monocytes into macrophages correlates with susceptibility to *L. major* infection in BALB/c versus B6 mice (19–21). Here, we addressed differentiation and functional phenotypes of inflammatory macrophages upon i.p. infection with *L. braziliensis* and *L. major* to investigate whether a maturation deficit reflects into an inability to develop protective M1 responses in BALB/c mice. We can summarize our results as follows:

1. Upon a transient recruitment of inflammatory monocytes by *L. braziliensis* infection, maturation into F4/80^int^ macrophages overcomes recovery of more mature F480^hi^ macrophages in BALB/c, but not B6 mice.
2. Replacement of immature Ly6C^+^F480^int^ monocytes by mature macrophages in B6 mice is accompanied by development of M1 macrophages, expressing more IL-12p35 and NOS2 and less arginase 1 than macrophages from BALB/c mice.
3. *L. braziliensis* infection increases macrophage maturation and induces a more robust M1 response in BALB/c mice than *L. major* parasites.

Altogether, these findings suggest that the development of protective M1 responses comes along with monocyte differentiation to mature macrophages and depends on both parasite infection and mouse strain.

More efficient induction of M1 responses by *L. braziliensis* might explain why unstimulated macrophages control better infection with *L. braziliensis in vitro*, whereas control of *L. major* infection requires activation by T-cell cytokines (33). Similarly, BALB/c mice control better cutaneous lesions upon *L. braziliensis* versus *L. major* infection (34). Moreover, a strong M1 response may underlie more severe clinical manifestations associated with inflammation in humans infected with *L. braziliensis* (17, 18). Finally, delayed and reduced macrophage maturation in BALB/c mice infected with *L. braziliensis* and *L. major* corroborate previous results (19–21) and seems to be associated with defective development of M1 responses.

As inflammatory monocytes mature into M1 macrophages, we investigated whether treatment with ATRA short upon *L. major* infection could affect the functional phenotype and anticipate induction of M1 macrophages. Treatment *in vivo* with ATRA, however, did not help development of M1 responses in BALB/c mice and, therefore, it did not rescue BALB/c macrophages to express effective immune responses. Moreover, ATRA impaired NOS2 expression as well as secretion of M1 cytokines, such as IL-12 and TNF-α by macrophages from *L. major*-infected mice and increased parasite burden in macrophages from BALB/c and B6 mice. Previously, we found that ATRA promoted the differentiation of inflammatory monocytes into mature macrophages *in vitro*, but also enhanced *L. major* replication within macrophages (23). Moreover, subcutaneous injection of ATRA reduced NO-mediated suppression of T-cell responses in B6 mice, but paradoxically fostered the development of footpad lesions and parasite load in draining lymph nodes (23). Likewise, supplementation with vitamin A increased parasite burden in the hamster model of visceral Leishmaniasis (35). Collectively, these results indicate that ATRA uncouples maturation and microbicidal activity of macrophages.

Here, we show that the effects of ATRA go beyond control of parasite infection, by affecting induction of M1-inflammatory macrophages. We tested this idea in the absence of *Leishmania* infection, by culturing BALB/c and B6 BMDMs under M1 plus M2 conditions in the presence of ATRA. We show that ATRA promoted an M1 to M2 transition in macrophages from both strains, by preventing expression of proinflammatory cytokines, NOS2, and NO, but increasing secretion of M2 chemokines. We also found a non-significant increase in arginase expression, which further reduces NOS2/arginase ratio. Similarly, ATRA and IL-4 synergize to induce arginase activity in macrophages and DCs (36–38), whereas the presence of LPS in our model may have antagonized this effect (36). Furthermore, expression of retinal dehydrogenase to produce retinoic acid seems to be part of the differentiation program of M2 macrophages and regulatory DCs (38–40).

Treatment with ATRA has been proposed as an anti-inflammatory therapy (38, 41, 42), as well as a tissue repair promoter, by affecting M1 and M2 macrophages or regulatory DCs (37, 41). Some reports also show direct inhibitory effects of ATRA on proinflammatory cytokine production by activated monocytes/macrophages (43–45). More studies are necessary to elucidate how ATRA affects M1/M2 macrophage phenotypes. ATRA may interfere with a shared signaling pathway, such as activation of NF-κB, which is necessary for multiple inflammatory responses (42). These results indicate that, independent of potential direct effects on adaptive immunity (24, 25), by targeting macrophages, ATRA might alter the nature of inflammation and induction of T-cell responses. Moreover, depending on how ATRA affects macrophage phenotype, treatment may improve immunity to tumors (9, 46) and extracellular pathogens (39, 47). Otherwise, treatment with ATRA can be detrimental for immunity to tumors (9) and intracellular parasites, such as *L. major*, which take advantage of M2 macrophages to proliferate in the absence of effective M1 responses.

Here, we show that whereas parasite-induced maturation may promote M1 phenotype in *Leishmania* infection, treatment with ATRA prevents differentiation into M1 macrophages and immunity to *L. major*. Further studies are necessary to address whether a therapy with ATRA could target exacerbated inflammatory responses to ameliorate pathology in mucocutaneous Leishmaniasis. In light of current findings, therapies based on treatment with ATRA should be evaluated with caution.

## Ethics Statement

All experiments were approved and conducted in accordance with guidelines of the Ethics Committee for Use of Animals, Federal University of Rio de Janeiro (UFRJ) (Protocol no. 078/16).

## Author Contributions

NV and SP-M performed and designed *in vivo* and *in vitro* experiments, analyzed data, and co-wrote the manuscript. NV, SP-M, MC-P, AF, FR-G, and TR performed cell culture, cytokine, and flow cytometry assays. GD contributed to interpretation of data and critical revision of the manuscript. ML designed the research, supervised the experiments, analyzed data, and wrote the manuscript. All authors approved the final version of the manuscript.

## Conflict of Interest Statement

The authors declare that the research was conducted in the absence of any commercial or financial relationships that could be construed as a potential conflict of interest.
