# Supplementary material for: All-Trans Retinoic Acid Promotes an M1- to M2-Phenotype Shift and Inhibits Macrophage-Mediated Immunity to Leishmania major
Source: Front Immunol. 2017 Nov 17;8:1560. doi: 10.3389/fimmu.2017.01560 (PMC5698282; doi:10.3389/fimmu.2017.01560)
Supplement: Supplementary file 1 [file data_sheet_1.docx]

Supplementary Material

All-*trans* retinoic acid promotes an M1 to M2-phenotype shift and inhibits macrophage-mediated immunity to *Leishmania major*

Natália S. Vellozo^1†^, Sâmara T. Pereira-Marques^1†^, Mariela P. Cabral-Piccin^1^, Alessandra A. Filardy^1,2^, Flávia L. Ribeiro-Gomes^1,3^, Thaís S. Rigoni^1^, George A. DosReis^1,4^, Marcela F. Lopes^1*^

^1^Instituto de Biofísica Carlos Chagas Filho, Universidade Federal do Rio de Janeiro, Rio de Janeiro, RJ, Brazil

^2^Instituto de Microbiologia Paulo de Góes, Universidade Federal do Rio de Janeiro, Rio de Janeiro, RJ, Brazil

^3^Instituto Oswaldo Cruz, Fundação Instituto Oswaldo Cruz, Rio de Janeiro, RJ, Brazil

^4^Instituto Nacional para Pesquisa Translacional em Saúde e Ambiente na Região Amazônica, Conselho Nacional de Desenvolvimento Científico e Tecnológico, Brazil

^†^These authors contributed equally to this work

*** Correspondence:** marcelal@biof.ufrj.br

# Supplementary Data

# Supplementary Figure 1 - Monocytes differentiate into macrophages upon *Leishmania* infection. PECs from control (A, left panels) B6 or (A and B) BALB/c mice, as well as from mice infected with (A) *L. braziliensis* or (B) *L. major* were evaluated for expression of Ly6C and F4/80 within CD11b^+^ gated cells. (A, B) Absolute numbers of F4/80^+^Ly6C^+^, F4/80^int^, and F4/80^hi^ cells from (A) *L. braziliensis*-infected B6 or BALB/c mice or (B) *L. major*-infected BALB/c mice compared with PECs from uninfected (d 0) mice. Each symbol represents individual control (□, N=3 or 6 mice/group) or infected mouse (■, N=5 mice/group). Means are represented. Significant differences were analyzed by ANOVA with Dunnett post-test and indicated for P<0.05 (*), P<0.01 (**), P<0.001 (***), and P<0.0001 (****).

Supplementary Figure 2 - Monocytes recruited upon i.p. infection with *L. major*. PECs from control BALB/c or B6 mice, as well as from mice infected 48 h before with *L. major* were evaluated for expression of Ly6C and F4/80 within CD11b^+^ cells. (A, B, C) Percentages of F4/80^+^Ly6C^+^, F4/80^int^, and F4/80^hi^ cells from *L. major*-infected BALB/c (Δ) or B6 (▼) mice compared with PECs from uninfected (ctl) mice. Each symbol represents individual control or infected (*Lm*) mouse (N=4 or 5 mice/group). Means are represented. Significant differences were analyzed by t-test and indicated for P<0.05 (*), P<0.01 (**), and P<0.0001 (****).

# Supplementary Figures

# Supplementary Figure 1 - Monocytes differentiate into macrophages upon *Leishmania* infection. PECs from control (A, left panels) B6 or (A and B) BALB/c mice, as well as from mice infected with (A) *L. braziliensis* or (B) *L. major* were evaluated for expression of Ly6C and F4/80 within CD11b^+^ gated cells. (A, B) Absolute numbers of F4/80^+^Ly6C^+^, F4/80^int^, and F4/80^hi^ cells from (A) *L. braziliensis*-infected B6 or BALB/c mice or (B) *L. major*-infected BALB/c mice compared with PECs from uninfected (d 0) mice. Each symbol represents individual control (□, N=3 or 6 mice/group) or infected mouse (■, N=5 mice/group). Means are represented. Significant differences were analyzed by ANOVA with Dunnett post-test and indicated for P<0.05 (*), P<0.01 (**), P<0.001 (***), and P<0.0001 (****).

**Supplementary Figure 2 -** Monocytes recruited upon i.p. infection with *L. major*. PECs from control BALB/c or B6 mice, as well as from mice infected 48 h before with *L. major* were evaluated for expression of Ly6C and F4/80 within CD11b^+^ cells. (A, B, C) Percentages of F4/80^+^Ly6C^+^, F4/80^int^, and F4/80^hi^ cells from *L. major*-infected BALB/c (Δ) or B6 (▼) mice compared with PECs from uninfected (ctl) mice. Each symbol represents individual control or infected (*Lm*) mouse (N=4 or 5 mice/group). Means are represented. Significant differences were analyzed by t-test and indicated for P<0.05 (*), P<0.01 (**), and P<0.0001 (****).
